# Advancing team-based primary health care: a comparative analysis of policies in western Canada

**DOI:** 10.1186/s12913-017-2439-1

**Published:** 2017-07-17

**Authors:** Esther Suter, Sara Mallinson, Renee Misfeldt, Omenaa Boakye, Louise Nasmith, Sabrina T. Wong

**Affiliations:** 1 Department of Social Work, University of Calgary, 2500 University Drive NW, Calgary, AB T2N 1N4 Canada; 2 Health Systems Evaluation and Evidence, Alberta Health Services, 10301 Southport Lane SW, Calgary, AB Canada; 3 Population, Public, and Indigenous Health, Alberta Health Services, 2210-2nd Street SW, Calgary, AB Canada; 4 University of British Columbia, 400-2194 Health Sciences Mall, Vancouver, BC Canada; 5 Centre for Health Services and Policy Research, University of British Columbia, 201-2206 East Mall, Vancouver, Canada

**Keywords:** Health policy, Policy analysis, Policy options, Primary care, Team-based care

## Abstract

**Background:**

We analyzed and compared primary health care (PHC) policies in British Columbia, Alberta and Saskatchewan to understand how they inform the design and implementation of team-based primary health care service delivery. The goal was to develop policy imperatives that can advance team-based PHC in Canada.

**Methods:**

We conducted comparative case studies (*n* = 3). The policy analysis included: *Context review*: We reviewed relevant information (2007 to 2014) from databases and websites. *Policy review and comparative analysis:* We compared and contrasted publically available PHC policies. *Key informant interviews:* Key informants (*n* = 30) validated narratives prepared from the comparative analysis by offering contextual information on potential policy imperatives*. Advisory group and roundtable:* An expert advisory group guided this work and a key stakeholder roundtable event guided prioritization of policy imperatives.

**Results:**

The concept of team-based PHC varies widely across and within the three provinces. We noted policy gaps related to team configuration, leadership, scope of practice, role clarity and financing of team-based care; few policies speak explicitly to monitoring and evaluation of team-based PHC. We prioritized four policy imperatives: (1) alignment of goals and policies at different system levels; (2) investment of resources for system change; (3) compensation models for all members of the team; and (4) accountability through collaborative practice metrics.

**Conclusions:**

Policies supporting team-based PHC have been slow to emerge, lacking a systematic and coordinated approach. Greater alignment with specific consideration of financing, reimbursement, implementation mechanisms and performance monitoring could accelerate systemic transformation by removing some well-known barriers to team-based care.

**Electronic supplementary material:**

The online version of this article (doi:10.1186/s12913-017-2439-1) contains supplementary material, which is available to authorized users.

## Background

In 2000, First Ministers — heads of governments including the Prime Minister of Canada and the provincial and territorial ministers— agreed that “improvements to primary health care are crucial to the renewal of health services” and highlighted the importance of multi-disciplinary teams [1]. The subsequent launch of the $800 M Primary Health Care Transition Fund was meant to accelerate innovation in primary health care delivery [2–4]. Initiatives have experimented with service delivery models that integrate physicians into interprofessional health care teams [3–6]. Recent reports applaud the progress made and conclude that primary health care has entered a period of transformational change [2–4, 6–9].

Despite these positive changes, Canada continues to lag behind other countries in primary health care infrastructure and performance [9, 10] and team-based primary health care remains fragmented [5]. Some have ascribed this to the provincial nature of health policy and the lack of a pan-Canadian vision on primary health care reform [3]. Others argue that the engrained ways in which primary care has historically been organized (e.g., physicians as independent business owners with direct billing to the provincial government for services provided) create significant barriers for team-based primary health care [11].

Primary health care reform remains a critical policy issue on national and provincial agendas [12]. For the purpose of this study, we defined health policies as the “decisions, plans, and actions undertaken to achieve specific health care goals within a society” [13]. Policy is driven by the unique political, social and economic contexts. The absence of a pan-Canadian vision for primary health care has led to different conceptualizations of team-based primary health care service delivery, the application of different policy levers, and, consequently, implementation of different policies across the province to support primary health care reform [4, 6, 10, 11].

It is unclear if and how current policy and regulatory landscapes advance or constrain progress towards team-based primary health care. There are few detailed examinations of the policy context for team-based primary health care including the key drivers of policy development and implementation [3].

The goal of this project was to develop policy imperatives to advance team-based primary health care in Canada. The objective was to analyze and compare relevant policies in three provinces to understand current policy landscape and gaps. The research question was: how do policies, including regulation and legislation in British Columbia, Alberta and Saskatchewan, inform the design and implementation of team-based primary health care service delivery? Sub-questions were: a) How is team-based care within primary health care conceptualized and defined within the policy documents, regulations and legislation? b) What are the common key approaches taken within policy documents (e.g., leadership, scope of practice, skill mix, role clarity, communication) that guide the implementation, oversight and administration of team-based primary health care service delivery? c) What policies, regulations and legislation are conducive to promoting team-based service delivery in primary health care in the three provinces?

## Methods

We conducted a comparative policy analysis of primary health care policies in three western Canadian provinces, British Columbia, Alberta and Saskatchewan, treating each province as a case study. These provinces were chosen because of the different and unique contexts in which team-based care was considered a policy priority. The policy triangle framework by Walt and Gilson guided our analysis of relationships and processes involved in primary health care policy across the provincial contexts [14] Additional file 1: Fig. S1: The health policy triangle.

The framework says that health policies are formed through the complex inter-play between the context, content, process, and actors involved [14]. Policy cannot easily be separated from health care politics, which comprises the interactions of political actors and institutions in the health care arena [12, 14]. Comparative analysis of the policy context within which health reform is formulated and implemented, as well as the processes involved, can contribute to strategies that increase the political feasibility of reform [15, 16]. Walt and Gilson’s framework is appropriate because it accounts for the complexity of primary health service delivery and the provincial health system context in which it takes place [17].

The policy triangle approach shaped the type of information we collected and our analysis. We needed to identify and acquire materials from diverse sources so we organized the review into several stages:

***Context review*** *:* We conducted a high-level scan of diverse evidence (review papers, reports, newspaper articles, editorials and other opinion pieces, briefings and book chapters) to examine the recent context of primary health care evolution nationally and in the three provinces. We searched Medline (through Pubmed), Google Scholar and the following websites Health Edition (*no longer operating), Health Council of Canada, Canadian Foundation for Healthcare Improvement, Enhancing Interdisciplinary Collaboration in Primary Health Care (EICP), Conference Board of Canada, Canadian Healthcare Association. Key words used in website searches were: “interprofessional”; “collaborative practice”; “collaboration”; “health care teams”; “Canada”; “Saskatchewan”; “Alberta”; “BC”; “British Columbia”; “primary care”; and “primary health care”. We systematically screened hits from the searches for inclusion using a brief appraisal tool to assess relevancy according to the following criteria: 1) Published between 2007/8–2013 (within 5 years); 2) Published in English; 3) *Substantial* focus on Canadian primary health care, that is, documents that described a national strategy and vision for primary health care, and/or focused on Alberta, British Columbia and Saskatchewan primary health care development, and/or focused on political, social and economic forces and primary health care reform in Canada or the three provinces.

We noted key events that occurred earlier but had impact that extend into our timeframe. The diverse information was collated into succinct narratives describing the national and provincial landscapes. We also developed visual provincial timelines to highlight a range of events that were shaping the development of primary health care and team-based care [18].

***Policy review and comparative analysis*** **:** We identified and retrieved publically available primary health care policies in British Columbia, Alberta and Saskatchewan. We only included formal policies (i.e., policies that are adopted by an organization and have authority to drive action) published between 2007 and 2014. Other supporting documents were considered as part of the context analysis [18]. We assigned one researcher to each province to initially extract information from retrieved policies into tables; a second researcher reviewed the policies and the extractions and edited or amended where needed. Reviewers resolved all disagreements by consensus discussions. The primary reviewer then developed concise narratives for each province, which were read and validated by the secondary reviewer. In a final step, the three reviewers compared and contrasted key findings and emerging themes from the three provinces, taking into account provincial context. Based on the analysis, we drafted potential policy imperatives.

***Key informant interviews*** **:** We invited key informants from the provincial ministries and departments of health, regional health authorities, primary health care organizations, and professional colleges and associations in each province to provide feedback on the provincial narratives and to offer additional information on local policy context and potential policy imperatives. The same three researchers that conducted the policy analyses also conducted the interviews for their respective province. Interviews lasted 45 to 60 min and were digitally recorded and transcribed. To begin the analysis process, 3–4 transcripts were independently read and marked by the research team. Early interpretations and ideas for themes were discussed and key themes and sub-themes were agreed. The transcript data were then summarized and re-ordered into thematic tables [19]. The research team regularly discussed progress to ensure shared understanding of the themes and appropriate interpretation of the data. A second reader checked the analysis of each transcript and any discrepancies in interpretation were resolved through team consensus.

***Advisory group and roundtable:*** We engaged an expert advisory group to guide the review and to take part in the identification and refinement of policy imperatives. A facilitated roundtable event was held with 15 representatives from the provincial ministries of health, regional health authorities, regulatory bodies and professional associations [20]. The goal was to further develop and prioritize the policy imperatives for action.

Ethics and operational approval was obtained from ethics board in each of the investigator’s universities and from the different health authorities.

## Results

Staying true to Walt and Gilson’s policy triangle [14], we reviewed our source materials with a view to context, content, process and actors involved in primary health care policy. The context review highlighted that all three provinces promote team-based primary health care guided by overarching frameworks and strategies. New structures have emerged that reflect the unique context of each province. For example, British Columbia invested in Integrated Health Networks and Divisions of Family Physicians; in Alberta the Primary Care Networks and Family Care Clinics emerged; and Saskatchewan implemented innovation sites. Political leadership changes often altered the focus of policies and primary health care structures.

We analyzed 45 policies from the three provinces (British Columbia *n* = 12, Alberta = 15, Saskatchewan *n* = 28) including strategy documents (e.g., charters, frameworks) and business plans from provincial ministries and health authorities (Table 1). Despite the unique provincial contexts and approaches, common issues and gaps emerged that will be further discussed below. These relate to stakeholder involvement in policy development, conceptualization of team-based primary health care and policy tools for implementation.

**Table 1:** Key policy documents cited in the manuscript^a^

British Columbia
BC1: British Columbia Ministry of Health. (2007). Primary Health Care Charter: A Collaborative Approach. Victoria, BC: British Columbia Ministry of Health. http://www.health.gov.bc.ca/library/publications/year/2007/phc_charter.pdf
BC2: British Columbia Government, British Columbia Medical Association & Medical Services Commission (2012). Physician Master Agreement. Retrieved from: http://www2.gov.bc.ca/assets/gov/health/practitioner-pro/medical-services-plan/pma-2012-consolidated-amendment-7.pdf
BC3: British Columbia Ministry of Health. (2013). Health Professions Act. Victoria, BC: British Columbia Queen’s Printer. http://www.bclaws.ca/Recon/document/ID/freeside/00_96183_01
BC4: British Columbia Ministry of Health. (2011). Bill 10–2011 Nurse Practitioners Statutes Amendment Act. Victoria, BC: British Columbia Ministry of Health. https://www.leg.bc.ca/39th4th/3rd_read/gov10-3.htm
Alberta
AB1: Alberta Health. (2014). Alberta’s Primary Health Care Strategy. Edmonton, AB: Alberta Health. http://www.health.alberta.ca/documents/Primary-Health-Care-Strategy-2014.pdf
AB2: Alberta Health. (2013a). Family Care Clinic Reference Manual. Edmonton, AB: Alberta Health. https://open.alberta.ca/publications/6859089
AB3: Alberta Health. (2013b). Family Care Clinic Governance and Accountability Guidelines. Edmonton, AB: Alberta Health. https://open.alberta.ca/publications/family-care-clinic-governance-and-accountability-guidelines
AB4: Alberta Health and Wellness, Alberta Medical Association Alberta Health Services (2008). Primary Care Initiative Policy Manual 10.1. Edmonton: Alberta Health and Wellness. Available at: https://www.pcnpmo.ca/access/Documents/PCN%20Policy%20Manual.pdf
AB5: Alberta Health and Alberta Health Services (2010). Becoming the Best: Alberta’s 5-Year Health Action Plan. Edmonton, AB: Alberta Health. https://open.alberta.ca/publications/9780778582861
Saskatchewan
SK1: Saskatchewan Ministry of Health. (2012). Patient Centred Community Designed Team Delivered: A Framework for Achieving a High Performing Primary Health Care System in Saskatchewan. Regina, SK: Saskatchewan Ministry of Health. https://www.saskatchewan.ca/~/media/files/health/additional%20reports/other%20ministry%20plans%20and%20reports/primary%20health%20care.pdf
SK2: Regina Qu’Appelle Health Region. (2008). Primary Health Care Strategic Plan 2008–2013. Regina, SK: Regina Qu’Appelle Health Region http://www.rqhealth.ca/programs/primary_healthcare/pdf_files/strategic_plan.pdf
SK3: Regina Qu’Appelle Health Region. (2013). Strategy for Touchwood Primary Health Care Collaborative. Regina, SK: Regina Qu’Appelle Health Region. http://www.rqhealth.ca/service-lines/master/files/rqhr_primary_care_strategy_touchwood.pdf
SK4: Government of Saskatchewan (2014). The Pharmacy and Pharmacy Disciplines Act. Chapter P-9.1 of the Statutes of Saskatchewan, 1996 (effective January 1, 1998). Regina: Queen’s Printer.
SK5: Government of Saskatchewan (2007) The Midwifery Act being Chapter M-14.1 of the Statutes of Saskatchewan, 1999 (effective February 23, 2007. Regina: Queen’s Printer.

^a^For a complete list of the policy documents we reviewed see Additional file 2

### Policies shaping team-based care: Development processes and stakeholder involvement

All three provinces position team-based primary health care in the wider context of primary care reform, underpinned by an overarching policy: British Columbia’s *Primary Health Care Charter* (BC1), Saskatchewan’s *Primary Health Care Framework* (SK1), and the recent *Primary Health Care Strategy* in Alberta (AB1). These documents set the vision for primary care reform with some notable differences. Saskatchewan’s *Framework* serves as a roadmap for the “where to” rather than the “how to” and allows teams to be configured based on local needs. Alberta’s *Primary Health Care Strategy* emphasizes an approach to team-based primary health care that integrates public health, wellness services, social services and community-based services (AB1). In contrast, British Columbia’s *Primary Health Care Charter* sets out a broad strategy for creating an effective and sustainable primary health care system with physicians firmly at the centre, supported by other providers (BC1).

The ministries of health together with the medical associations were the driving forces behind policy development in the three provinces. Saskatchewan, and more recently Alberta and British Columbia, engaged a broader range of stakeholders (providers, regulatory and education sectors) through working and advisory groups (BC1, AB1, SK1). We noted a lack of patient advocacy representation in policy development across the three provinces despite a patient-centred philosophy.

### The conceptualization and composition of primary health care teams

Policies include limited guidance on team composition and the organization of teams, such as team leadership roles or lines of accountability. Notable exceptions are the policies for Family Care Clinics in Alberta (AB2, AB3), which set minimum requirements for teams whilst still allowing flexibility to address community needs. Many policies ascribe (implicitly or explicitly) team leadership to family physicians (BC1, AB1). In addition, much of the funding for team-based primary health care is funneled through physicians (e.g., General Practice Services Committee in British Columbia (BC2), Primary Care Networks in Alberta (AB4), underscoring physician leadership and accountability. Some policies in Saskatchewan offer more flexibility as there is no requirement to have a physician as a team member or leader of the team (SK1). This is also the case in recent policies around team-based care for Alberta’s Family Care Clinics (AB2, AB3), which a physician or a nurse practitioner can lead.

A key concept emerging from the policies is the ability of all team members to work to full scope of practice. Saskatchewan’s *Framework* states that primary health care teams flourish when providers work to their full scope of practice (SK1). Alberta’s *Primary Health Care Strategy* notes under the principle of collaboration that members will work to full scope of practice and with defined roles and responsibilities (AB1). Neither policy mentions role clarity issues that might ensue from overlapping scope of interprofessional team members or how to resolve them. Policies are also silent on team liability.

### Policy tools to advance the implementation of team-based primary health care policies

We found a significant number of policies from health authorities that speak to team-based care (e.g., AB5, SK2, SK3). These policies ranged widely in level of detail and the extent to which they offer implementation guidance. With a few exceptions (e.g., AB3), most policy documents do not provide direction on tools that could be used to facilitate implementation of team-based care.

Some policies contained quality improvement goals to advance team-based primary health care (SK1). Quality improvement was framed in general terms through recommendations for using evidence-based quality improvement practices but with unclear links to team-based care. Other tools related to workplace culture. For example, in Alberta’s *Primary Health Care Strategy*, a key goal is to enhance collaborative practice and this is reinforced by reference to standards and frameworks (AB1). The Family Care Clinic policy documents (AB2, AB3) cited the Alberta *Collaborative Practice and Education Framework for* Change [21] as a tool to support collaboration and team-based care.

Policies in all three provinces discuss funding primary health care transformation, although the type and direction of investments vary in scale and focus. In some cases, investments are explicitly short-term to attract and retain family physicians in places with demonstrated needs. Such was the case in British Columbia where family physicians received targeted funds to expand their primary care service offerings. Most policies did not discuss funding to support the introduction of team-based care models (e.g., through education or mentorship programs), enhanced infrastructure for shared electronic health records or workspace for teams. There was also little evidence within the policies of long-term investments to sustain culture and practice changes.

Although remuneration is an important consideration in primary health care redesign, few policies make explicit reference to payment models. A notable exception is the Alberta *Primary Health Care Strategy* (AB1), which includes the improvement and alignment of compensation models as a strategic goal (AB1). Funding and incentives to attract and retain physicians are mentioned in British Columbia’s *Primary Health Care Charter* (BC1).

Another potential mechanism for change is enhancing scopes of practice or introducing new provider roles. Scope of practice was expanded for Nurse Practitioners in British Columbia (BC3) and pharmacists in Alberta (AB5) and Saskatchewan (SK4); Saskatchewan also introduced regulated midwifery services (SK5). The regulatory changes allow existing and new providers to assume more comprehensive roles in primary health care. However, none of the policies were explicitly intended as a mechanism to advance team-based primary health care. One possible exception is the Health Professions Act in BC, which “urges” collaboration between providers (BC4).

Lastly, a number of key policies outline accountability and performance monitoring criteria and processes (AB1, SK1) there is, however, no common accountability framework or consistent approach to measuring outcomes of team-based primary health care models. Some policies merely suggest the need for evaluation or plans to develop an evaluation framework (e.g. BC1, AB2); Alberta’s *Primary Health Care Strategy* has an evaluation logic model that includes the assessment of interdisciplinary collaborative care, although specific metrics are not elaborated on (AB1).

### Stakeholder feedback

Thirty representatives from health ministries, regional health authorities and professional organizations (BC *n* = 9, AB *n* = 10, SK *n* = 11) commented on the draft provincial policy narratives. Overall, key informants felt that the narratives were accurate.

- It was noted that there are many good examples of team-based care but that there was a lack of ways to share these examples; many commented on the limited operational guidance or practice resources to support implementation of team-based care.
- Almost every key informant raised concerns about the distribution and control of funding streams, which may hinder progressive policy development. They argued that it is essential to finding better ways to remunerate all the members of primary health care teams and fund team development.
- Stakeholders stressed the importance of moving beyond collaborative practice as a strategic goal and the need for appropriate investment in change management to overcome years of inertia. The need for infrastructure (collaborative space, IT) and education for collaborative practice was a recurrent theme.
- Informants gave illustrations of the ways in which poor alignment of policies and practices across the health system impedes the development of team-based care.

The key informant interviews helped us understand the context in which certain policies were developed and how they were implemented on the ground. Informants also shared valuable insights on potential policy imperatives.

### Advisory group and roundtable feedback

The research team identified a long list of policy imperatives from the policy synthesis and stakeholder interviews. After review and individual ranking by our research advisory team (based on perceived relevancy and feasibility), four policy imperatives rose to the top. These included alignment of goals and policies at different system levels, investment of resources for system change, compensation models for all members of the team and accountability through collaborative practice metrics. Roundtable participants identified the key elements for each of the four policy imperatives and the inherent conflicts and potential trade-offs that may arise [20]. Further discussions focused on the most important outcomes and critical implementation factors. Table 2 describes the final policy imperatives thought to be feasible and effective in advancing team-based primary health care service delivery.

**Table 2:** Policy imperatives for advancing team-based primary health care

Policy imperative #1: To align health system goals, policies, workforce and structures
The lack of system alignment between the ministries of health, regional health authorities and private practices’ priorities and agendas impedes team-based care. To align health system goals, policies, workforce and structures requires a shared vision on team-based care, resource sharing and inclusion of the broader community in policy deliberation and implementation. We urge leaders to stay the course for team-based primary health care as it is foundational to health care reform in Canada.
Policy imperative #2: To invest adequate resources to support system change structures
Provinces need to invest adequate resources to support system change to foster team-based primary health care. This requires that provinces provide sustainable funding for team-based care and invest in proper infrastructure, adequate technology and change management plans. Improved evidence is required to guide decisions on resource assignment.
Policy imperative #3: To develop appropriate and sustainable compensation models
Existing compensation models can negatively impact team-based service delivery. The third policy imperative is therefore to develop appropriate and sustainable compensation models. This imperative is not directed solely at physician remuneration; we need to consider compensation models for a wider range of team members including those in the community.
Policy imperative #4: To integrate collaborative practice metrics in primary health care monitoring and evaluation structures
The fourth policy imperative, integrate collaborative practice metrics in primary health care monitoring and evaluation, will improve accountability for team-based service provision. Investments need to be made in shared data elements and indicators so we can learn from successes and failures, target our investments and disinvest in initiatives that do not yield the desired outcomes.

## Discussion

### The policy landscape

More than a decade ago, the First Ministers agreed to team-based care as a cornerstone of primary health care renewal in Canada [1]. This comparative policy analysis examined how existing key policies in British Columbia, Alberta and Saskatchewan speak to team-based primary health care and support or hinder progress. Based on our policy triangle approach and analysis, which includes the contextual review, comparisons of policies, validation of policy narratives with key informants and the advisory group and roundtable feedback, we make the following observations about the policy landscape.

*Lack of details on implementation and evaluation:* Many of the current publicly available policy frameworks reference team-based primary health care, but how team-based care is conceptualized and defined varies widely. They also lack details on team configuration, leadership, scope of practice and role clarity; there is seldom reference or guidance on the policy tools (e.g., investments in education or mentorship programs) that could facilitate collaboration by health care providers. We also note that few policies speak explicitly to monitoring and evaluation of the new service delivery models. These significant policy gaps may hamper full implementation of team-based care.

Watson and Wong, based on their critical policy and context review of the Enhancing Interdisciplinary Collaboration in Primary Health Care Initiative (EICP), made a similar observation 10 years earlier [22]. They concluded at the time that until a national vision for team-based primary health care exists and permeates regulatory frameworks and policies, it will be difficult to advance team-based care on the ground. Deber and Baumann echoed this statement by suggesting that provincial regulatory frameworks for interprofessional collaboration are needed urgently, with particular attention to the division of responsibilities between provincial governments and professional regulatory bodies [23]. They further suggested that attention be paid to the scope of practice for each profession, how scope is defined across jurisdictions and the distinct and overlapping competencies of different providers. There has been much movement over the past years with new legislative policies redefining the scope of registered nurses, nurse practitioners and pharmacists in several provinces, elevating their role in team-based primary health care [24, 25]. These legislative changes can work to accelerate reform if leveraged properly. However, some have argued that these roles have not always been introduced with sufficient articulation of how they will be integrated into existing service delivery models or how they will impact the scopes of practice of other health professions [25]. Also, these innovations require careful evaluation of outcomes associated with different team-based models of care. There is consensus in the literature that clear reporting expectations must exist for continuous improvement of team-based care but current policies offer limited guidance in that respect [2, 9, 23, 24, 26].

*Funding issues:* Current policies do not adequately address the funding of team-based primary healthcare. Funding pertains to two distinct areas: firstly, targeted, evidence-based and sustainable funding and resource allocation for infrastructure, change management plans, educational programs and training, and adequate technology to facilitate collaboration; and secondly, changes in compensation models for all health care providers (including those in the community) to incentivize team-based care. Both issues have been extensively discussed in the literature as key barriers to the adoption of team-based care [2, 9, 10, 22–25]. The primary health care reform agenda was supported by hefty, albeit time limited investments from provincial and federal levels that created the impetus for new service delivery models [10, 24]. However, some argue that not enough resources were allocated to training and coaching for culture change to overcome entrenched social values that act as barriers to team-based care [9]. Also, in the absence of clear funding guidance and regulatory changes, many jurisdictions have maintained their two-stream system where health authorities and physicians have different funding structures and accountabilities. It has long been recognized that fee for service remuneration is incompatible with the development of interprofessional teams in primary health care and that payment models need to be reformed to align with overall health system goals [24, 27–30].

*Lack of policy evolution and alignment within and across systems:* Our analysis across three provinces would suggest that policies during this period to support team-based primary health care have been slow to emerge and have lacked a systematic and coordinated approach across sectors and geographic areas. More importantly, the policies lacked alignment in the strategic planning for primary health care between provincial Ministries of Health, regional health authorities and clinics. Alignment pertains to a clearly articulated vision and consistent messages, realignment of ministry of health, regional health authorities and private practices’ priorities and agendas, and alignment of the acute care/primary care interface. In his examination of health reform across western countries, Hacker elaborates on the relationship between governance structure, financing and health reform. He argues that in political structures, such as in Canada, where implementation of health reform is under provincial jurisdiction, “…rapid or decisive structural policy change has proved far more elusive” (p694) [31]. He further points out that countries like Canada with many veto points (introduced by federalism, powerful judiciary, distributed administrative and political responsibility for planning), may be more prone to policy stalemate as the political system makes change difficult. In such constellations, the federal Government lacks the capacity to formulate objectives and monitor progress, and practice change occurs within existing policy frameworks. Ettel et al. make a similar observation in comparing health planning in New Zealand and Germany. They comment on the fragmented planning in Germany, attributed to federalism and corporatism, which has resulted in great diversity in response [31]. In contrast, due to the central authority of the government in health care planning in New Zealand, planning is relatively coherent and promotes alignment across sectors and geographical boundaries.

Several authors have noted that primary health care reform in Canada has been primarily implemented voluntarily, based on incentives [9, 24]. Much of the change has been negotiated, largely preserving the autonomy of physicians [6, 9]. Many new models are criticized as being limited and lacking the characteristics of high performing models, having only partial interdisciplinarity and remaining physician-centred [9, 32].

### Policy imperatives

The main goal of this research was to identify policy imperatives to advance team-based primary health care. We used our observations about commonalities and gaps in existing policies, stakeholder interviews, and advisory group priority ranking to arrive at four policy imperatives:

- To align health system goals, policies, workforce and structures,
- To invest adequate resources to support system change structures,
- To develop appropriate and sustainable compensation models,
- To integrate collaborative practice metrics in primary health care monitoring and evaluation structures.

The roundtable discussion, at which key health system representatives discussed relevance and feasibility, strongly affirmed the policy imperatives [20]. Participants argued against prioritizing them as they saw the policies as equally important and interconnected. Initially termed policy options, they recommended to use the term policy “imperatives” to avoid the risk of “cherry-picking” that might occur if presented as options. There was consensus at the end of the day that swift action is required and that collectively, the four policy imperatives have the potential to advance team-based primary health care across Canada. The next step, which was beyond the scope of this project, is to engage the provinces in a detailed review of implementation considerations for these imperatives.

### Limitations

This work was not without challenges, many of them methodological. Conducting searches of diverse evidence, agreeing on criteria for inclusion, and developing extraction tools and ways to validate information all required some creativity. Testing policy research methods and ways of engaging stakeholders is vital to support evidence-based policy development. Another challenge is the ever-evolving nature of policy within the three provinces throughout our project period. We acknowledge that our research took a snapshot of policies within a specific timeframe. Nevertheless, the four policy imperatives emerging from this policy synthesis remain relevant and may constitute the cornerstones for a pan-Canadian framework.

## Conclusion

Hacker’s analysis of health care reform has referred to a paradoxical pattern of policy reform that he described as “reform without change and change without reform” (p721) [31]. The latter seems befitting for the way team-based primary health care has evolved across the three western provinces and across Canada. There is evidence of pockets of innovations that fit unique contexts in the absence of overarching policy reform across levels. Using a policy triangle lens for our policy review helped to focus our inquiry on the relationship between context and policy evolution in different places and what this may mean for national policy development [14].

Through this comparative policy study, we identified four policy imperatives that speak to the gaps and limitations evident in the current policies in three Canadian provinces. These policy imperatives could present a mechanism to advance team-based primary health care without stifling local innovation. The four policy imperatives are interrelated and need to be incorporated into policies as a total package to achieve system wide reform. Given the federalist nature of the Canadian health care system, some believe that a national vision for team-based primary health care might be elusive and local context and power dynamics will continue to drive policy solutions [31, 33]. We argue, however, that pushing the policy frameworks towards greater alignment with specific consideration of financing, reimbursement, implementation mechanism and performance monitoring could accelerate progress by removing some well-known barriers to team-based care. Based on our research, we concur with recent reports that urged the Canadian governments at all levels to make a shared commitment to scale up innovations and create coordinated systems of funding, financing, remuneration and education that enable team-based models of care that align with patient outcomes and optimal scopes of practice [2, 25].

## Additional files

Additional file 1: Contains a full list of policy documents reviewed in this study. (DOCX 21 kb)

Additional file 2: Contains an diagram of the health policy triangle (DOCX 27 kb)
