# Supplementary material for: Advancing team-based primary health care: a comparative analysis of policies in western Canada
Source: BMC Health Serv Res. 2017 Jul 17;17:493. doi: 10.1186/s12913-017-2439-1 (PMC5512982; doi:10.1186/s12913-017-2439-1)
Supplement: Supplementary file 1 — Contains a full list of policy documents reviewed in this study. (DOCX 21 kb) [file 12913_2017_2439_MOESM1_ESM.docx]

**Content**

**Context**

**Process**

**Actors**

- Individuals
- Groups
- Organizations
