# Supplementary material for: Advancing team-based primary health care: a comparative analysis of policies in western Canada
Source: BMC Health Serv Res. 2017 Jul 17;17:493. doi: 10.1186/s12913-017-2439-1 (PMC5512982; doi:10.1186/s12913-017-2439-1)
Supplement: Supplementary file 2 — Contains an diagram of the health policy triangle (DOCX 27 kb) [file 12913_2017_2439_MOESM2_ESM.docx]

Additional file 1: Policy Documents Included in Analysis

Alberta

Alberta Health. (2012). Family Care Clinic Application Kit: Wave 1. Edmonton, AB: Alberta Health. Retrieved from <http://www.health.alberta.ca/services/family-care-clinic-about.html>

Alberta Health. (2013b). Health Business Plan 2013 – 2016. Edmonton, AB: Alberta Health. Retrieved from <http://www.finance.alberta.ca/publications/budget/budget2013/health.pdf>

Alberta Health. (2013c). Family Care Clinic Reference Manual. Edmonton, AB: Alberta Health. Retrieved from <http://www.health.alberta.ca/documents/PHC-FCC-Reference-Manual.pdf>

Alberta Health. (2013d). Family Care Clinic Governance and Accountability Guidelines. Edmonton, AB: Alberta Health. Retrieved from [http://www.health.alberta.ca/documents/PHC-Family Care Clinic-Governance-Guidelines.pdf](http://www.health.alberta.ca/documents/PHC-FCC-Governance-Guidelines.pdf)

Alberta Health. (2013e). Family Care Clinic Workforce Guidelines. Edmonton, AB: Alberta Health. Retrieved from [http://www.health.alberta.ca/documents/PHC-Family Care Clinic-Workforce-Guidelines.pdf](http://www.health.alberta.ca/documents/PHC-FCC-Workforce-Guidelines.pdf)

Alberta Health. (2014a). Alberta’s Primary Health Care Strategy. Edmonton, AB: Alberta Health. Retrieved from <http://www.health.alberta.ca/documents/Primary-Health-Care-Strategy-2014.pdf>

Alberta Health and Alberta Health Services. (2010). Becoming the Best: Alberta’s 5-Year Health Action Plan. Edmonton, AB: Alberta Health. Retrieved from <http://www.health.alberta.ca/documents/Becoming-the-Best-2010.pdf>

Alberta Health Services. (2011). Health Plan 2011-2015. Edmonton, AB: Alberta Health Services. Retrieved from <http://www.albertahealthservices.ca/publications/ahs-pub-2011-2015-health-plan.pdf>

Alberta Health Services. (2012). 2012-2015 Strategic Direction. Edmonton, AB: Alberta Health Services. Retrieved from <http://www.albertahealthservices.ca/MissionStrategicDirection/ahs-msd-strategic-direction-2012-2015.pdf>

Alberta Health & Wellness, Alberta Medical Association & Alberta Regional Health Authorities. (2006a) PCI Guide for Other Health Care Providers. Edmonton, AB: Alberta Health Retrieved from [www.albertapci.ca](http://www.albertapci.ca) (no longer available)

Alberta Health & Wellness, Alberta Medical Association & Alberta Regional Health Authorities (2006b) PCI Program Evaluation Framework. Edmonton, AB: Alberta Health. Retrieved from [www.albertapci.ca](http://www.albertapci.ca)  [(no longer available)](http://www.albertapci.ca/OperatingPCN/Pages/Default.aspx)

Alberta Health & Wellness, Alberta Medical Association & Alberta Regional Health Authorities. (2006c). PCI Business Plan Template. Edmonton, AB: Alberta Health. Retrieved from <http://www.albertapci.ca/OperatingPCN/developingapcn/Documents/16.a)BusinessPlanTemplate.doc>

Alberta Health and Wellness, Alberta Medical Association & Alberta Health Services (2008). Primary Care Initiative Policy Manual 10.1. Edmonton, AB: Alberta Health and Wellness. Retrieved from <http://www.albertapci.ca/OperatingPCN/Documents/Policies%20and%20Manuals/PCIPolicyManual.pdf>

Government of Alberta (2010). Alberta Health Act. Edmonton, AB: Alberta Queen’s Printer. Retrieved from <http://www.qp.alberta.ca/1266.cfm?page=A19P5.cfm&leg_type=Acts&isbncln=9780779754809>

Government of Alberta (2013). Health Professions Act: Revised Statutes of Alberta 2000. Edmonton, AB: Alberta Queen’s Printer. Retrieved from <http://www.qp.alberta.ca/documents/acts/h07.pdf>

British Columbia

British Columbia Ministry of Health. (2007). Primary Health Care Charter: A Collaborative Approach. Victoria, BC: British Columbia Ministry of Health. Retrieved from <http://www.health.gov.bc.ca/library/publications/year/2007/phc_charter.pdf>

British Columbia Ministry of Health. (2012). 2012/13 – 2014/15 Service Plan. Victoria, BC: British Columbia Ministry of Health. Retrieved from <http://www.bcbudget.gov.bc.ca/2012/sp/pdf/ministry/hlth.pdf>

British Columbia Ministry of Health. (2014). 2014/15 – 2016/17 Service Plan. Victoria, BC: British Columbia Ministry of Health. Retrieved from [www.bcbudget.gov.bc.ca/2014/sp/pdf/ministry/hlth.pdf](http://www.bcbudget.gov.bc.ca/2014/sp/pdf/ministry/hlth.pdf).

British Columbia Government, British Columbia Medical Association & Medical Services Commission. (2012). Physician Master Agreement. Retrieved from <http://www.health.gov.bc.ca/msp/legislation/pdf/pma-consolidated-amendment-7.pdf>

British Columbia Ministry of Health. (2013b). Health Professions Act. Victoria, BC: British Columbia Queen's Printer. Retrieved from <http://www.bclaws.ca/Recon/document/ID/freeside/00_96183_01> (no longer available)

Fraser Health Authority. (2012). 2012/13 – 2014/15 Service Plan. Surrey, BC: Fraser Health Authority.

Interior Health Authority. (2012). 2012/13 – 2014/15 Service Plan. Kelowna, BC: Retrieved from <http://www.interiorhealth.ca/AboutUs/Accountability/Documents/Service%20Plan%202012-13_2014-15.pdf>

Northern Health Authority. (2009b). Strategic Plan 2009-2015. Prince George, BC: Retrieved from <http://www.northernhealth.ca/Portals/0/About/Strategic%20Plan/Stategic%20Plan%20Brochure.pdf>

Vancouver Coastal Health Authority. (2012). 2012/13 – 2014/15 Service Plan. Vancouver, BC: Vancouver Coastal Health Authority. Retrieved from <https://www.vch.ca/media/Service-Plan-2012-2012_Vancouver-Coastal-Health.pdf>

Vancouver Island Health Authority. (2012). 2012/13 – 2014/15 Service Plan. Victoria, BC: Vancouver Island Health Authority. Retrieved from <http://www.viha.ca/NR/rdonlyres/AADEEF61-F0F4-47F8-B65B-268D2F6D1FDB/0/HSP_2008_2009.pdf>

Vancouver Island Health Authority. (2009a). Primary Health Care Strategy Framework Refresh (2009/10 – 2012/13). Victoria, BC: Vancouver Island Health Authority: Retrieved from <http://www.viha.ca/NR/rdonlyres/453A2276-E33E-400E-82A7-F618D65A922B/0/PHCStrategyFinal.pdf> (no longer available)

Vancouver Island Health Authority. (2009b). Five-Year Strategic Plan 2008-2013. Victoria, BC: Vancouver Island Health Authority: Retrieved from <http://www.viha.ca/NR/rdonlyres/0496C63E-96FE-4A36-852F-20E408AA02AB/0/strategic_plan_2009.pdf>

Saskatchewan

Cypress Health Region Strategic Plan. (no date). Strategic Direction. Swift Current, SK: Cypress Health Region Strategic Plan. Retrieved from <http://www.cypresshealth.ca/page.php?id=155>

Five Hills Health Region. (2013). Strategic Plan 2013 -2014. Moose Jaw, SK: Five Hills Health Region. Retrieved from <http://www.fhhr.ca/Documents/2013-14StrategicPlanFHHR-BoardApproved.pdf>

Heartland Health Region. (2007). Strategic Plan 2007 – 2010. Rosetown, SK: Heartland Health Region. Retrieved from <http://www.hrha.sk.ca/documents/Strat%20Plan%20April%201%202007.pdf>

Kelsey Trail Health Region. (2010). Strategic and Operations Directions. Tisdale, SK: Kelsey Trail Health Region. Retrieved from <http://www.kelseytrailhealth.ca/Publications/KTHRPlans/Documents/KTHR%202010-11%20Strategic%20Directions%20and%20Appendices%2008-10.pdf> (no longer available)

Prairie North Health Region. (2007). Strategic Plan 2007 – 2010. North Battleford, SK: Prairie North Health Region. Retrieved from <http://www.pnrha.ca/bins/doc.asp?rdc_id=1125>

Prairie North Health Region. (2010). Strategic Framework & Plan 2010 – 2013. North Battleford, SK: Prairie North Health Region. Retrieved from <http://www.pnrha.ca/bins/doc.asp?rdc_id=4702>

Prince Albert Parkland Health Region. (2010). Strategic Plan 2010-2014. Prince Albert, SK: Prince Albert Parkland Health Region. Retrieved from <http://www.princealbertparklandhealth.com/OnlinePublications/PublicationsOnline_download.asp?L936_=GBTEPublication&fieldname=File1path&where=+%5BPublicationsOnline%5D%2E%5BID%5D%3D358>

Regina Qu'Appelle Health Region. (2008). Primary Health Care Strategic Plan 2008 – 2013. Regina, SK: Regina Qu'Appelle Health Region. Retrieved from <http://www.rqhealth.ca/programs/primary_healthcare/pdf_files/strategic_plan.pdf>

Regina Qu'Appelle Health Region. (2009). Indian Head District Primary Health Care Plan. Regina, SK: Regina Qu’Appelle Health Region. Retrieved from <http://www.rqhealth.ca/programs/primary_healthcare/pdf_files/indian_head.pdf#xml=http://www.rqhealth.ca/cgibin/texis.cgi/webinator/search_rhd/+Fw5zmxwww/xml.txt?query=Indian+Head+District+Primary+Health+Care+Plan+&pr=rqhr&order=r&cq=&id=52cebddb2b>

Regina Qu'Appelle Health Region. (2013a). Strategy for Touchwood Primary Health Care Collaborative. Regina, SK: Regina Qu’Appelle Health Region. Retrieved from <http://www.rqhealth.ca/programs/primary_healthcare/pdf_files/rqhr_primary_care_strategy_touchwood.pdf>

Regina Qu'Appelle Health Region. (2013b). Strategic Plan 2013 – 2017. Regina, SK: Regina Qu’Appelle Health Region. Retrieved from <http://www.rqhealth.ca/> (no longer available)

Regina Qu'Appelle Health Region. (2013c). Business Plan 2013-2014. Regina, SK: Regina Qu'Appelle Health Region. Retrieved from <http://www.rqhealth.ca/> (no longer available)

Regina Qu'Appelle Health Region. ( no date ). Regional Health Workforce Plan 2012 – 2014. Regina, SK: Retrieved from <http://www.rqhealth.ca/inside/publications/workforce_plan/workforce_plan_2012_2014.pdf>

Saskatchewan Ministry of Health. (2009). Health Plan 2009 – 2010. Regina, SK: Saskatchewan Ministry of Health. Retrieved from <http://www.finance.gov.sk.ca/PlanningAndReporting/2009-10/HealthPlan0910.pdf>

Saskatchewan Ministry of Health. (2010). Plan for 2010 – 2011. Regina, SK: Saskatchewan Ministry of Health. Retrieved from <http://www.finance.gov.sk.ca/PlanningAndReporting/2010-11/HealthPlan1011.pdf>

Saskatchewan Ministry of Health. (2011a). Saskatchewan’s Health Human Resources Plan. Regina, SK: Saskatchewan Ministry of Health. Retrieved from <http://www.health.gov.sk.ca/adx/aspx/adxGetMedia.aspx?DocID=5c66f3a8-899f-4d35-9456-72b5487caa4c&MediaID=5579&Filename=sask-health-human-resources-plan.pdf&l=English>

Saskatchewan Ministry of Health. (2011b). Plan for 2011 -2012. . Regina, SK: Saskatchewan Ministry of Health. Retrieved from <http://www.finance.gov.sk.ca/PlanningAndReporting/2011-12/HealthPlan1112.pdf>

Saskatchewan Ministry of Health. (2012a). Patient Centred Community Designed Team Delivered: A Framework for Achieving a High Performing Primary Health Care System in Saskatchewan. Regina, SK: Saskatchewan Ministry of Health. Retrieved from <http://www.health.gov.sk.ca/phc-framework-report>

Saskatchewan Ministry of Health. (2012b). Plan for 2012 – 2013. Regina, SK: Saskatchewan Ministry of Health. Retrieved from <http://www.finance.gov.sk.ca/PlanningAndReporting/2012-13/HealthPlan1213.pdf>

Saskatchewan Ministry of Health. (2013b). Plan for 2013 – 2014. Regina, SK: Saskatchewan Ministry of Health. Retrieved from <http://www.finance.gov.sk.ca/PlanningAndReporting/2013-14/HealthPlan1314.pdf>

Saskatchewan Ministry of Health and Health Care Systems. (2014). Plan for 2014 – 2015. Regina, SK: Saskatchewan Ministry of Health. Retrieved from <http://www.finance.gov.sk.ca/PlanningAndReporting/2014-15/HealthPlan1415.pdf>

Saskatchewan Ministry of Health & Saskatchewan Medical Association. (2011a). Letter of Understanding. Retrieved from <http://www.health.gov.sk.ca/sma-lou-2011>

Saskatchewan Ministry of Health & Saskatchewan Medical Association. (2011b). Payment Schedule. Retrieved from <http://health.gov.sk.ca/sma-agreement-2011>

Saskatoon Health Region. (2012a). Strategic Plan 2010 – 2013. Saskatoon, SK: Saskatoon Health Region. Retrieved from <http://www.saskatoonhealthregion.ca/documents/Strategic-Plan-2010-2013-vision-mission-directions.pdf> (no longer available)

Saskatoon Health Region. (2012b). Workforce Action Plan 2012 – 2016. Saskatoon, SK: Saskatoon Health Region. Retrieved from <http://www.saskatoonhealthregion.ca/about_us/documents/workforce_action_plan_2012.pdf>

Saskatoon Health Region. (2013). Primary Health Care Progression Plan 2013. Saskatoon, SK: Saskatoon Health Region.

Sun Country Health Region. (2012). Strategic Plan 2012 – 2013. Weyburn, SK: Sun Country Health Region**.** Retrieved from <http://www.google.ca/url?sa=t&rct=j&q=&esrc=s&frm=1&source=web&cd=1&ved=0CCkQFjAA&url=http%3A%2F%2Fwww.suncountry.sk.ca%2FgsCMSDisplayPluginFile%2Fshow%2Fid%2F380%2Fmenu_id%2F160%2Flang_type%2Fen_US%2Fpage_type%2Fpeople%2Fpage_id%2F31&ei=5x_QUvydCpDsoASmyoKICQ&usg=AFQjCNFZhkN7zyTh1bnqiNahB20X-5d07Q&bvm=bv.59026428,d.cGU>

Sunrise Health Region. (2012). Strategic Plan 2012 – 2017. Yorkton, SK: Sunrise Health Region. Retrieved from <http://www.sunrisehealthregion.sk.ca/images/Articles/28_SHR%20Strategic%20Plan%202012-2017%20Final.pdf>
